# Salon nail care with superficial self-disclosure vitalizes psychological state

**DOI:** 10.3389/fpsyg.2023.1112110

**Published:** 2023-09-19

**Authors:** Atsushi Kawakubo, Takashi Oguchi

**Affiliations:** ^1^Department of Psychology, Faculty of Humanities, Saitama Gakuen University, Kawaguchi, Japan; ^2^Department of Psychology, College of Contemporary Psychology, Rikkyo University, Saitama, Japan

**Keywords:** attractiveness, nail care, psychological effect, makeup, self-disclosure, Japanese

## Abstract

Physical attractiveness has long been established as a desirable trait in society. Physically attractive individuals are considered more competent, successful, and sociable. Numerous studies have examined the influence of makeup on physical attractiveness and its subjective psychological effects. However, the effect of nail care has not yet been examined. This study aimed to explore the psychological effects of nail care. Female Japanese participants (*N* = 334) completed an online questionnaire. The results showed that nail care positively elevated three aspects: positive emotions, relaxation, and vitalization. Moreover, they confirmed significant differences between receiving nail care from salon manicurists and self-performed nail care in terms of positive emotions and relaxation. The results indicated that nail care altered the appearance of the recipients’ nails and their self-esteem, including their feelings, mood, and motivation. Furthermore, this study indicates that it may be preferable for both manicurists and clients not to self-disclose in-depth, as this would negate the positive effects of nail care. The study concludes with recommendations for future research.

## 1. Introduction

People have expressed beauty in numerous ways in recent years. In pursuit of “beauty,” which is not limited to outward appearance, many seek essential care, health, eco-friendly lifestyles, and overall happiness. By engaging consumers with beauty-related products, industries maintain and expand their markets (Pudaruth et al., 2015).

### 1.1. Makeup enhances physical attractiveness

Anthropologists emphasize the importance of physical appearance variation in developing personal and social relationships (Korichi et al., 2008). For instance, classical studies have revealed that physically attractive individuals are more competent, successful, and socially adept than less attractive individuals (e.g., Berscheid and Walster, 1974; Goldman and Lewis, 1977). In the early stages of many relationships, physical attractiveness is an essential determinant of interpersonal attraction, providing others with easily accessible, nonverbal information about a person (e.g., Nakdimen, 1984).

Apart from interpersonal relationships, physical attractiveness has other varied effects. Nielsen and Kernaleguen (1976) suggested that facial attractiveness influences social satisfaction, social desirability, and the subjective assessment of overall physical attractiveness more than bodily attractiveness. Moreover, from an evolutionary perspective, facial attractiveness is a vital indicator of good genes and well-being, a signal for friendship choice (Thornhill and Gangestad, 1999), and influences perceived leadership ability (e.g., Van Vugt and Grabo, 2015).

Physical attractiveness is essential to humans; therefore, it is crucial to consider ways to enhance attractiveness. Commercial cosmetics are among the most prevalent strategies used to enhance the perceived attractiveness of women’s faces. In modern society, women applying makeup is considered a daily ritual. Many cosmetics in the market artificially modify the appearance of facial features, enabling women to conform to feminine beauty standards; for example, they enhance the visual impact of the eyes and lips, narrow the eyebrows, and redden the cheeks (Korichi et al., 2008). Many women wear facial makeup daily to accentuate their attractiveness, make a good impression, enhance their features, and portray a youthful appearance.

Previous studies have established that cosmetics positively affect attractiveness, and several others have shown that facial images with makeup are considered more physically attractive (e.g., Cash et al., 1989; Mulhern et al., 2003; Jones et al., 2018). For instance, makeup significantly and positively influences the evaluation of a woman’s face. At first glance and over extended inspection periods, competence ratings increase significantly with makeup (Etcoff et al., 2011). According to Bielfeldt et al. (2013), cosmetic treatments, such as cleansers and light foundations, can alter women’s facial attractiveness. Similarly, Tagai et al. (2016) examined the influence of light (natural) and heavy (glamorous) makeup on attractiveness ratings and facial recognition. Their research demonstrated that light makeup is preferable to heavy makeup because it does not impede facial recognition and produces a positive impression on the beholder. Moreover, the most recent findings indicate that makeup enhances women’s attractiveness, perceptions of competence, and feelings of warmth (Kellie et al., 2021).

Applying makeup may also be helpful in romantic situations. Makeup allows romantically-motivated women to attract potential partners by increasing their perceived dominance and prestige (Mileva et al., 2016). When attracting and competing for a desired romantic partner, women may benefit from applying makeup to improve their appearance (Davis and Arnocky, 2022). Similarly, Mafra et al. (2020) proposed that women apply makeup to alter their appearance to make themselves appear younger, more attractive, and have better partners than their rivals.

### 1.2. Makeup’s psychological effects

Given the consistent findings that cosmetics beautify the face, researchers have also investigated the subjective psychological changes associated with wearing makeup. Previous studies suggest that makeup can alter a person’s mental state and outward appearance. Miller and Cox (1982) demonstrated that public, but not private self-consciousness is systematically related to variations in facial cosmetic use. Private and public self-consciousness constructs are based on the direction of focus and attention, that is, whether they are directed inward (internal feelings and beliefs about oneself) or outward (beliefs about the opinions of others; DaSilveira et al., 2015). Miller and Cox (1982) reported that publicly self-conscious women tend to wear more makeup because they believe that it enhances their appearance. Additionally, Uyama et al. (1990) reported that the psychological benefits of cosmetics include improved proactiveness, relaxation, and positive mood.

Sato (2020) conducted an Internet survey of 2,340 Japanese women aged 20–44 years to investigate cosmetics’ impact on people’s minds. To this end, the survey examined women’s feelings and attitudes toward 10 cosmetics categories. The results revealed that the effects of makeup in cosmetics include emotional factors, such as “relaxation/refreshment” and “energy/vigor.” Moreover, the experimental results of Japanese participants showed that the psychological effects of makeup boosted satisfaction and self-confidence. Makeup makes people more self-confident, leads them to a relaxed emotional state, and positively affects their mental health and well-being (Yogo et al., 1990). Tran et al. (2020) cited improved confidence as a benefit of makeup use and described it as a means to enhance or manipulate appearance and mood.

Beyond merely applying colorful products to the face, there has been consistent evidence that makeup manifests as a holistic technique to modify one’s appearance and assists with self-image, emotions, and mood. According to Korichi et al. (2008), makeup application could be a daily routine. It can improve the positive aspects related to an individual’s self-image and social environment and lessen the negative aspects.

Furthermore, some studies have examined the effects of makeup on psychological interventions. Richard et al. (2019) investigated the immediate, short-term, and midterm effects of a beauty care intervention on depression symptoms, quality of life, self-esteem, and body image in patients with early-stage breast cancer. The intervention consisted of a single-session group workshop on makeup, after which the participants reported a reduction in depressive symptoms, improvement in quality of life, and higher self-esteem. Similarly, Park et al. (2015) examined psychological factors, including distress, self-esteem, and sexual function, before and after cosmetic interventions. Compared to the control group, participants in the treatment group relied significantly less on stress management and avoidance but not on self-esteem.

### 1.3. Cosmetic treatment for nails

Several studies have investigated the influence of makeup on physical attractiveness and its subjective effects. However, to the best of our knowledge, only a few studies on nail care have explored these effects using scientific or statistical methods. One of the few findings is that makeup and nail care enhance the human body, with the belief that smooth, glossy, and attractive nails symbolize health and youth; therefore, they are highly desirable (Chang et al., 2007). Both makeup and nail care adorn the body. Thus, it can be inferred that both produce similar psychological effects. However, the difference is that makeup is applied to the face, and nail care is performed on the fingers. In addition, people cannot see facial makeup without a mirror, whereas nails can be easily seen.

The nail cosmetics industry continues to expand to meet growing consumer demand. Continuous changes in fashion trends and beauty awareness have fuelled the growth of the nail care market. In 2017, the United States market volume reached approximately $8.5 billion, although growth has slowed recently (Nails Magazine, 2018). Currently, women and men practice regular nail care worldwide. People with healthy nails are better able to accomplish their work and enjoy their busy lives (Reinecke and Hinshaw, 2020). The novelty of this study lies in its exploration of the subjective effects of nail care from a psychological perspective.

### 1.4. Self-disclosure in nail salons

There is a unique feature of nail care not found in facial cosmetics. Nail care is often applied by professionals in specialized salons, as opposed to facial cosmetics, which are mostly self-applied. In 2017, approximately 56,000 salons in the United States offered nail care services on a daily basis (Nails Magazine, 2018). There is an inevitable connection between the customers and practitioners at these salons. This study examined the relationship between salon communication, that is, self-disclosure to manicurists, and the psychological effects of nail care on women.

Self-disclosure involves revealing personal information to others, thereby gaining their sympathy (Collins and Miller, 1994). Building relationships between frontline employees and customers is increasingly emphasized (Andersson et al., 2016), and salespeople’s self-disclosure fosters long-term customer relationships (e.g., Crosby et al., 1990). Thus, it is crucial for businesses to establish good relationships between frontline employees and customers.

Moreover, in a customer service environment, frontline employees’ satisfaction reflects the customer’s experience (e.g., Jacobs et al., 2001). Ganesh et al. (2000) reported that if an employee forms a positive rapport with a customer, the customer is likely to feel positively inclined toward the employee, which converts to mutual satisfaction. Therefore, this study predicts that self-disclosure in nail salons positively influences the subjective effects of the services received.

### 1.5. Research objectives

This study investigated the psychological effects of receiving nail care and communicating with the practitioner. First, we examined the effects of nail care based on past literature concerning the effects of wearing makeup. Second, we determined whether there is a difference between the subjective effects of nail care when self-applied versus when applied by manicurists in professional salons. This led us to hypothesize that the effects of nail care vary broadly depending on the person performing the application (self-application or expert application).

*Hypothesis 1*: Compared with self-application, expert application of nail care enhances subjective psychological effects.

Additionally, we examined the effects of communication between customers and manicurists.

*Hypothesis 2*: Manicurists’ profound self-disclosure enhances the subjective psychological effects of nail care.

## 2. Method

Using an online survey, we recruited Japanese women from various age groups and regions (urban and local areas). All participants were recruited through Rakuten Insight, a leading online market research company in Japan.

Internet surveys have often been scrutinized for reliability, as online survey respondents tend to devote insufficient attention to answering questions and respond without carefully reading the instructions or questions (Miura and Kobayashi, 2015). Therefore, to increase the reliability of the answers, an item was added to the questionnaire: “Please select not at all.” The analysis targeted respondents who answered this item correctly.

The participants comprised 500 Japanese women whose ages ranged from early 20s to late 30s, with a mean age of 30.64 years (*SD* = 4.78). All participants resided in metropolitan areas (Tokyo and three surrounding provinces). They used smartphones or computers to complete the questionnaire.

The participants’ age, residence, household income, marital status, items related to nail care (e.g., single nail care fees and nail salon visits per month), monthly cosmetics expenses, and the items below were obtained via demographic survey questions (note that “nail care” refers to painting nails, such as coloring, nail art, and gel nails).

### 2.1. Information on nail care

First, the participants were asked whether they frequented nail salons. This question was designed to separate respondents into three groups: (1) those who received nail care from salon manicurists; (2) those who self-applied nail care without visiting a nail salon; and (3) those who had neither been to a nail salon nor painted their nails.

### 2.2. Psychological effects of nail care

Based on the items measuring the psychological effects of makeup used by Uyama et al. (1990), we developed items measuring the psychological effects of nail care. Participants were asked to identify altered attitudes or emotions caused by nail care. They were instructed to “Select the appropriate response that best indicates how much you typically experience each item listed below during a manicure.” The items were evaluated on a four-point scale ranging from one (not at all) to four (a great deal).

We used the 24-item scale developed by Niwa and Maruno (2010) to measure the depth of self-disclosure in nail salons. The scale has a high sensitivity for reflecting four varying degrees of self-disclosure in terms of participants’ hobbies and daily life (Level I, e.g., “What I am passionate about?”), difficult experiences (Level II, e.g., “What is the most painful event I have ever experienced?”), disadvantages and weaknesses (Level III, e.g., “What are my insurmountable obstacles?”), and negative personality and abilities (Level IV, e.g., “What constitutes a terrible part of my personality?”).

The participants were instructed as follows: “Recall a typical conversation with a manicurist at a nail salon. Write a number next to each statement to reflect the extent to which you discussed that topic with that manicurist.” Only those who received nail care from a salon manicurist responded to these items. On a seven-point scale, ranging from one (not at all) to seven (very detailed), participants were asked to report the extent to which they discussed each item with the manicurist.

The Japanese sample’s internal consistency reliability indices ranged from 0.86 to 0.87 in the study by Niwa and Maruno (2010). In this study, the reliability indices included hobbies and daily life (Level I; α = 0.92), difficult experiences (Level II; α = 0.94), disadvantages and weaknesses (Level III; α = 0.97), and negative personality and abilities (Level IV; α = 0.97). We used the mean values of the items related to each level for analysis. This study inferred that the subjective psychological effects of nail care would be more remarkable when a customer communicates more with a salon manicurist (the more they self-disclose). Therefore, a comprehensive model was developed to capture the relationship between two latent variables, namely the depth of self-disclosure and the psychological effects of nail care.

## 3. Results

This study investigated the psychological effects of receiving nail care and examined communication with a practitioner. Therefore, the analysis excluded respondents who belonged to Group 3 (those who had never visited a nail salon or painted their nails). Thus, we analyzed the data of 334 women with a mean age of 30.70 years (*SD* = 4.66). A total of 167 participants were included in the salon-nail group (those who received nail care from salon manicurists; mean age = 30.57 years, *SD* = 4.39) and those in the self-nail group (those who self-applied nail care without going to a nail salon; mean age = 30.83 years, *SD* = 4.92). Table 1 presents the descriptive statistics for each group. Using the chi-square test to analyze whether there was a significant difference between the annual income of the two groups, we found no significant differences when the analysis was conducted after grouping into one group with annual incomes of JPY10,000,000 or more [χ^2^(5) = 7.79, *ns*]. Hence, we assumed no income effects on the differences between the groups in our subsequent analysis.

**Table 1:** Descriptive statistics for each sample group.

Variables	Group 1 (*n* = 167)		Group 2 (*n* = 167)	
	*n*	%	*n*	%
Household income				
Under 400	39	23.4	54	32.3
401–600	35	21.0	37	22.2
601–800	31	18.6	22	13.2
801–1,000	18	10.8	18	10.8
1,001–1,200	7	4.2	8	4.8
1,201–1,500	10	6.0	3	1.8
More than 1,500	13	7.8	6	3.6
Refused to answer	14	8.4	19	11.4
Marriage status				
Married	74	44.3	68	40.7
Never married	87	52.1	94	56.3
Divorced	6	3.6	5	3.0
	*M*	*SD*	*M*	*SD*
The expense for cosmetics (JPY/month)	18214.11	17366.75	12763.50	13460.74
The charge for a nail-care at a salon (JPY)	7093.89	3616.16	—	—
Number of nail salon visits(month)	1.2	0.6	—	—

The currency unit of household income is JPY 10,000.

This study included a series of analyses that adhered to the objectives and predetermined procedures. First, the factor structure of items pertaining to the psychological effects of nail care was examined. Second, the results of the salon-nail and self-applied nail care were compared. Third, we explored the relationship between the effects of nail care on Japanese women and self-disclosure to salon manicurists.

### 3.1. Exploratory factor analysis of the psychological effects of nail care

Using a maximum likelihood estimator with promax rotation, an exploratory factor analysis (EFA) was performed on 30 items to examine the factor structure of the psychological effects of nail care. In the social sciences, correlations among factors are typically expected since behavior is rarely partitioned into neatly packaged units that function independently of one another (Costello and Osborne, 2005). Thus, we employed a promax rotation, which is an oblique rotation rather than an orthogonal rotation. Although the EFA extracted five factors with eigenvalues greater than 1 (Kaiser, 1960), a parallel analysis (O’connor, 2000), minimum average partial (MAP) test (Velicer, 1976), and visual scree plot analysis (Zoski and Jurs, 1996) were performed to establish the optimal and reliable number of factors. The results revealed three factors with eigenvalues of 13.55, 3.09, and 1.57. Based on the recommendations of Henson and Roberts (2006), a three-factor model was employed that used multiple criteria to determine the number of factors and avoid overdependence on the eigenvalues >1 rule.

Additionally, whether items were to be retained was determined based on two criteria (Worthington and Whittaker, 2006): (a) the factor loadings were less than 0.32, and (b) absolute loadings were higher than 0.32 for two or more factors. Considering the eigenvalues of the factors and the remaining items, five items that did not meet these criteria were omitted. Finally, the three-factor model accounted for 65.65% of the common variance, yielding a significant Bartlett’s test of sphericity (*p* < 0.01) and a Keiser-Meyer-Olkin value of 0.95. Both methods confirmed that sufficiently large relationships exist within the dataset of interest to perform an EFA (Howard, 2016). Based on these results, we inferred that the three-factor model is optimal. The final EFA results are presented in Table 2.

**Table 2:** Results of the three-factor EFA (*n* = 334).

			I	II	III
I: Positive Emotions (α = 0.95, 14 items)					
23.	Happy		**0.97**	−0.11	−0.14
25.	Enjoyable		**0.87**	−0.04	−0.07
8.	Excited		**0.85**	−0.24	0.11
9.	Lively		**0.83**	−0.23	−0.02
26.	Refreshing		**0.81**	0.08	−0.03
20.	Feeling pleasant		**0.69**	0.22	−0.06
24.	Love myself		**0.69**	0.09	−0.01
28.	Feminine		**0.65**	−0.16	0.25
21.	Relieve stress		**0.63**	0.19	−0.07
22.	Bright		**0.63**	0.27	−0.02
18.	Cheerful		**0.57**	0.19	0.14
19.	Motivated		**0.56**	0.25	0.08
17.	My face cleared		**0.52**	0.22	0.17
27.	Become youthful		**0.47**	0.13	0.20
II: Relaxation (α = 0.92, 7 items)					
11.	To be free		−0.04	**0.93**	−0.09
12.	Relax		−0.01	**0.92**	−0.14
14.	Be kind		−0.15	**0.82**	0.17
13.	Become calm		−0.07	**0.81**	0.08
16.	Become peaceful		0.01	**0.81**	−0.09
15.	Refreshing		−0.06	**0.73**	0.10
29.	feel easy		0.16	**0.58**	−0.01
III: Vitalization (α = 0.82, 4 items)					
3.	Want to go outside		0.02	−0.03	**0.87**
1.	Want to meet people		0.17	−0.18	**0.79**
2.	Want to do something		−0.10	0.16	**0.68**
7.	Become positive		0.15	0.25	**0.35**
	Factor correlation				
		I	—	0.58	0.69
		II		—	0.45
		III			—

Factor loadings above 0.35 are in bold.

### 3.2. Variations of effects by a nail care practitioner

Hypothesis 1 was considered after confirming the factor structure of the effects of nail care. Compared to self-application, receiving nail care services from salon manicurists enhanced its subjective psychological effects. We examined the differences in the effects between the two groups, that is, the variations in experience between those who received nail care services from salon manicurists and those who self-applied their nail care. Table 2 shows that the three factors of nail care effects (positive emotions, relaxation, and vitalization) were significantly correlated. A multivariate analysis of variance was performed.

**Figure 1:**
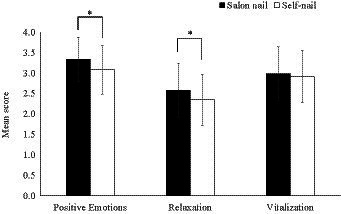
Variations between groups in the mean scores of nail care’s effects (*p < 0.01). The error bars represent the standard deviation of the measurement.

There were significant multivariate main effects of the group (Wilks’s Λ = 0.93, *F* [3, 330] = 8.86, *p* < 0.01, η_p_^2^ = 0.08). Furthermore, there were significant univariate main effects for the groups with positive emotions (*F* [1, 332] = 17.73, *p* < 0.01; η_p_^2^ = 0.05) and relaxation (*F* [1, 332] = 11.11, *p* < 0.01; η_p_^2^ = 0.03). However, there was no significant difference among the groups in terms of vitalization (*F* [1, 332] = 1.11, *ns*; η_p_^2^ = 0.00). Figure 1 indicates that the scores for positive emotions and relaxation in the salon-nail group were significantly higher (*p*s < 0.01) than those in the self-nail group. These results partially support the hypothesis that receiving nail care from salon manicurists leads to more significant subjective psychological effects than self-application.

### 3.3. Influence of self-disclosure at nail salons

In the subsequent analysis, only Group 1 was included because it specifically examines the relationship between the depth of self-disclosure and the psychological effects of nail care. Correlation coefficients between variables are shown in Table 3.

**Table 3:** Descriptive statistics and correlations of variables (Group1; *n* = 167).

		*M*	*SD*	1	2	3	4	5	6	7
1	Positive emotions	3.34	0.54	—						
2	Relaxation	2.58	0.66	0.60^**^	—					
3	Vitalization	2.99	0.66	0.67^**^	0.48^**^	—				
4	Self-disclosure: Level I	4.23	1.42	0.14	0.19^*^	0.16^*^	—			
5	Self-disclosure: Level II	2.10	1.34	−0.07	0.12	−0.02	0.52^**^	—		
6	Self-disclosure: Level III	2.12	1.33	−0.01	0.12	0.07	0.50^**^	0.84^**^	—	
7	Self-disclosure: Level IV	1.76	1.13	−0.14	0.06	−0.01	0.42^**^	0.82^**^	0.87^**^	—

* *p* < 0.05.

** *p* < 0.01.

**Figure 2:**
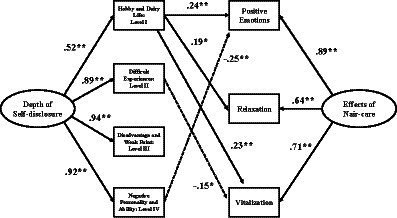
Model showing the relationship between the constructs of depth of self-disclosure and nail care’s effects (*n* = 167; *p < 0.01, **p < 0.001). χ^2^ = 17.22, 9 degrees of freedom (*p* < 0.05), CFI = 0.988, RMSEA = 0.074, SRMR = 0.030. Numbers indicate standardized path coefficients and dotted lines indicate significant negative effects.

Three goodness-of-fit (GOF) measures were employed to assess the proposed model’s compatibility with the data. A comparative fit index (CFI) measured the relative fit by comparing the proposed model to a null model, implying no common variances among the analyzed items, with greater values indicating a better model fit. Measures of absolute fit included (1) the root mean square error of approximation (RMSEA), which indicated the average discrepancy in model fit per degree of freedom, with smaller values indicating a better model fit; and (2) a standardized root mean square residual (SRMR), which indicates the absolute value of the standardized fitted residuals’ average size, with smaller values indicating a better model fit. In confirming GOF, CFI > 0.90 (Bentler and Bonett, 1980), RMSEA < 0.08 (Browne and Cudeck, 1993), and SRMR < 0.08 (Hu and Bentler, 1998) represented an acceptable model fit. Figure 2 demonstrates that the standard path coefficients and significance levels reveal the relationships between the constructs of depth of self-disclosure and the psychological effects of nail care.

These findings confirmed a significant relationship between the two latent variables. Superficial self-disclosure (hobbies and daily life) was positively associated with positive emotions (β = 0.24, *p* < 0.01), relaxation (β = 0.19, *p* < 0.05), and vitalization (β = 0.23, *p* < 0.01). In contrast, higher levels of self-disclosure negatively impacted the effects of nail care. Specifically, difficult experiences significantly negatively impacted vitalization (β = −0.15, *p* < 0.05), and negative personality and ability negatively affected positive emotions (β = −0.25, *p* < 0.01). These results revealed that those who received nail care perceived various subjective psychological effects depending on the depth of communication with the manicurist. Therefore, Hypothesis 2, which states that deeper self-disclosure with practitioners enhances the subjective psychological effects of nail care, was not supported.

## 4. Discussion

This study examined the psychological effects of nail care and determined the relationship between these effects and self-disclosure to practitioners. The desire to enhance one’s appearance is embedded in one’s social and daily life. Social and scientific research on the psychological effects of nail care on people’s consciousness is growing. Therefore, gathering credible evidence regarding people’s appearances is crucial to contribute to the current literature. This study makes a new contribution to psychological research in various ways.

First, the psychological effects of cosmetics were replicated by confirming that nail care had several effects on positive emotions, relaxation, and vitalization. People experience happiness and gratification when their nails are being manicured. Simultaneously, nail care effectively calms and relaxes the receiver. People who receive nail care feel more extroverted, prefer being in public and interacting with others. These results such as increased positivity, relaxation, and positive mood were similar to the psychological effects of using cosmetics reported in previous studies (Uyama et al., 1990; Sato, 2020), confirming that the psychological effects of nail care are consistent with those of makeup. Given the practical and theoretical implications for psychological science, our results expand previous research on the effects of cosmetics and confirm that the same holds true for nail care.

Second, this study demonstrates that compared to those who self-administer nail care, people who receive nail care from salon manicurists experience a more substantial effect on positive emotions and relaxation. The number of people caring for their nails continues to increase, and the practice has become widespread. Moreover, the latest statistics (Nails Magazine, 2018) indicate that the number of manicurists is increasing. This study demonstrated the influence on the subjective effects of nail care performed at dedicated places. Despite the financial expense, one of the reasons people visit nail salons is that our fingertips are often the most visible parts of the body. Unlike facial cosmetics, nail care can be performed without a mirror, using only one’s eyes. Considering these nail care characteristics, our results suggest that salon nail care may effectively improve an individual’s motivation and mood.

Third, testing the model revealed statistically significant positive and negative associations between depth of self-disclosure and the psychological effects of nail care. Depth of self-disclosure regarding negative personality and ability had a negative effect on positive emotions. Conversely, the self-disclosure of hobbies and daily life positively affected each of the three psychological effects of nail care. People attempt to convey their thoughts, information, feelings, and experiences to others daily. In the early stages of a relationship, self-disclosure typically begins superficially by disclosing one’s preferences and hobbies. As intimacy with the recipient increases, the level of self-disclosure deepens to include worries and personal shortcomings (Altman and Taylor, 1973). This study shows that self-disclosure between customers and manicurists is more effective when not in-depth. Based on these results, manicurists must understand the mechanism of self-disclosure to add value to their services and distinguish themselves from their competitors. This study proposes that receiving nail care at a salon with superficial self-disclosure may be more effective than self-performed nail care.

Although we aimed to conduct an accurate analysis of the research questions, several limitations must be noted. First, because the current study lacked a control group, we could not make rigorous comparisons regarding the effects of nail care. Ideally, it would have been necessary to collect responses from participants with no nail care and compare those with data from participants with nail care. As the scale used measured the subjective state of nail care, it was impossible to collect data for the control group. It is essential to acknowledge the potential impact of selection bias owing to the absence of a control group, which may have selectively included individuals with a considerable interest in nail care for the analysis. Selection bias refers to the bias that arises when groups or data are chosen for analysis in a manner that does not achieve proper randomization, leading to inaccurate results (Hernán et al., 2004).

Second, participants’ self-reported results of the questionnaires were analyzed to assess the psychological effects of nail care. Therefore, the exact causal relationship among these effects must be considered. Establishing causality is a principal challenge and has been a central area of psychological research in recent years. Holland and Rubin (1988) noted that “statistics can establish correlation, but not causation”; hence, it is worth noting that this study was correlational, and the directionality of effects cannot and should not be inferred from the data.

Third, there is no guarantee that the analyzed data are entirely representative. The participants in this study were Japanese women of a certain age (early 20s to late 30s) residing in metropolitan areas. Makeup has diverse meanings and preferences across different cultures. Therefore, caution should be exercised when extrapolating the results to other nationalities or age groups. Verifying our results will be a task for future research. Additionally, confounding factors that affected the results, such as participants’ annual income, were not fully controlled. Furthermore, this study focused only on one aspect of communication, namely, the depth of self-disclosure. For a more comprehensive understanding, future studies should also investigate the influence of the self-discloser’s personality and similarities in sociodemographic attributes (e.g., age and gender) among self-disclosers.

Finally, there is a direction of research that examines the psychological effects of nail care on individuals’ temporary mood during application and holistic welfare. In this study, the psychological effects of nail care measures were limited to general sentiments rather than feelings on a particular day (e.g., immediately after receiving nail care at a salon). Consequently, our analysis was not dependent on reports obtained immediately after nail care at the salon. Future research should apply experimental procedures or examine longitudinally whether nail care is associated with personal well-being and motivation in daily life. This study, despite its shortcomings, is the beginning of research on nail care. Therefore, further research is anticipated.

## Data availability statement

The raw data supporting the conclusions of this article will be made available by the authors, without undue reservation.

## Ethics statement

The studies involving humans were approved by College of Contemporary Psychology, Rikkyo University. The studies were conducted in accordance with the local legislation and institutional requirements. Written informed consent for participation was not required from the participants or the participants’ legal guardians/next of kin in accordance with the national legislation and institutional requirements.

## Author contributions

AK analyzed data and wrote the first draft of the manuscript. TO supervised the project and critically reviewed the manuscript. All authors contributed to the article and approved the submitted version.

## Conflict of interest

The authors declare that the research was conducted in the absence of any commercial or financial relationships that could be construed as a potential conflict of interest.

## Publisher’s note

All claims expressed in this article are solely those of the authors and do not necessarily represent those of their affiliated organizations, or those of the publisher, the editors and the reviewers. Any product that may be evaluated in this article, or claim that may be made by its manufacturer, is not guaranteed or endorsed by the publisher.
